# Targeting Hsp70 Immunosuppressive Signaling Axis with Lipid Nanovesicles: A Novel Approach to Treat Pancreatic Cancer

**DOI:** 10.3390/cancers17071224

**Published:** 2025-04-04

**Authors:** Ahmet Kaynak, Subrahmanya D. Vallabhapurapu, Eric P. Smith, Harold W. Davis, Clayton S. Lewis, Joseph Ahn, Petr Muller, Borek Vojtesek, Keith F. Stringer, Robert S. Franco, Vladimir Y. Bogdanov, Wen-Hai Shao, Xiaoyang Qi

**Affiliations:** 1 Division of Hematology & Oncology, Department of Internal Medicine, College of Medicine, University of Cincinnati, Cincinnati, OH 45267, USA; kaynakat@ucmail.uc.edu (A.K.); subrahmaya@gmail.com (S.D.V.); smithep4554@gmail.com (E.P.S.); harold.davis19@gmail.com (H.W.D.); lewis2c3@ucmail.uc.edu (C.S.L.); jha9504@gmail.com (J.A.); francors@ucmail.uc.edu (R.S.F.); 2 Masaryk Memorial Cancer Institute, Research Centre for Applied Molecular Oncology, Zluty Kopec 7, 656 53 Brno, Czech Republic; pmuller@post.cz (P.M.); vojtesek@mou.cz (B.V.); 3 Division of Pathology and Laboratory Medicine, Cincinnati Children’s Hospital Medical Center, Cincinnati, OH 45229, USA; keith.stringer@cchmc.org (K.F.S.); 4 Center for Scientific Review, National Institutes of Health, Bethesda, MD 20892, USA; (formerly at University of Cincinnati) vladimir.bogdanov@nih.gov (V.Y.B.); 5 Division of Rheumatology, Allergy & Immunology, Department of Internal Medicine, College of Medicine, University of Cincinnati, Cincinnati, OH 45267, USA; shaowi@ucmail.uc.edu (W.-H.S.)

**Keywords:** cancer cell-secreted Hsp70, phosphatidylserine, M2 macrophage polarization, dioleoylphosphatidylglycerol **(**DOPG**)**, Saposin C-coupled dioleoylphosphatidylglycerol (SapC-DOPG), immunotherapy, targeting therapy, pancreatic cancer

## Abstract

Background: Despite many efforts to effectively treat PDAC, PDAC carries one of the highest mortality rates of all major cancers. Thus, there is a critical unmet need to develop novel approaches to improve the clinical outcome of PDAC. It is well known that many cancers, including PDAC, generate a local TME that allows cancer to escape normal immune surveillance. Phosphatidylserine (PS), a negatively charged phospholipid that is abundant on the cancer cell membrane and with known actions to promote the secretion of immunomodulatory proteins, may provide a mechanism to regulate the TME. This study explored that possibility. Methods: MΦ differentiation and polarization were assessed by Western blotting and flow cytometric approaches. PS exposure and surface markers were analyzed by flow cytometry. Protein–protein and protein–lipid interactions were analyzed by immunofluorescence and enzyme-linked immunosorbent assay (ELISA). Phospholipid and SapC-DOPG treatment were employed to assess target protein functions in MΦ polarization, tumor growth, and survival in subcutaneous and orthotopic tumor models. The PK-PD and safety of SapC-DOPG were tested on orthotopic mouse models. Results: Our studies show that PDAC secretes Hsp70 that stimulates the MΦ polarization to the immunosuppressive M2 phenotype. We found that high surface PS on cancer cells correlates with increased secretion of Hsp70 and is associated with higher MΦ differentiation activity in vitro and in vivo. Furthermore, blocking cancer cell-secreted Hsp70 with SapC-DOPG reverses the immune suppression and reduces tumor growth. Conclusions: These preclinical results reveal a novel immunotherapeutic approach to potentially improve the outcome of PDAC treatment in humans.

## Simple Summary

Pancreatic ductal adenocarcinoma (PDAC) is the twelfth most frequent cancer worldwide and the fourth leading cause of cancer-related deaths in the USA, and is projected to be ranked second by 2030. FOLFIRINOX or gemcitabine-abraxane, the current standard treatments for advanced PDAC, prolong survival by only several months in chemo-sensitive patients. Thus, there remains an urgent medical need to develop novel approaches to treat PDAC. Since the PDAC tumor microenvironment (TME) is highly immunosuppressive with M2 polarized macrophages (MΦ), inhibition of immunosuppressive M2 polarization offers a potentially robust approach to reactivate the immune system against PDAC. In this study, we developed a Saposin C (SapC)-coupled dioleoylphosphatidylglycerol (SapC-DOPG) that binds secreted heat shock protein 70 (Hsp70), which was recently shown to be secreted by PDAC cells and to be a potent mediator of TME immunosuppression. Our preclinical studies using mouse models of pancreatic cancer show that SapC-DOPG blocks Hsp70 actions and inhibits tumor growth. These preclinical studies suggest a promising immunotherapeutic approach to treat PDAC.

## 1. Introduction

Cancer cells have evolved mechanisms to establish an immunosuppressive tumor microenvironment (TME) that promotes escape from host immune surveillance, resulting in sustained tumor growth [1,2,3,4]. Reversal of this immunosuppression is an increasingly employed modality for cancer treatment [1,3,5,6]. The TME is composed of a variety of cells, soluble factors, and tumor-associated macrophages (TAMs). TAMs are mostly immunosuppressive M2 polarized macrophages (MΦs) derived from resident MΦs.

Recent studies have demonstrated that the inhibition of M2 MΦ polarization through interleukin (IL)-27 leads to a reduction in the proliferation, migration, and metastatic capacity of pancreatic cancer cells in vitro [7]. Furthermore, colony stimulating factor 1 receptor (CSF-1R) inhibitors, which selectively target CSF-1R, and are predominantly expressed by monocytes and macrophages, result in reduced M2 MΦ polarization and enhanced survival in a pancreatic ductal adenocarcinoma (PDAC) mouse model [8]. Therefore, strategies that block the development of immunosuppressive M2 TAMs and facilitate immune activation against the tumor provide a promising therapeutic approach for pancreatic cancer. However, increasing the effectiveness of this approach will require (1) the identification of cancer cell-derived factors that regulate M2 MΦ polarization and (2) approaches to therapeutically block identified mediators.

Despite decades of efforts to develop effective therapies against PDAC, it continues to manifest high morbidity and mortality; ~90% of patients diagnosed with PDAC succumb within five years of diagnosis [9]. The resistance to treatment with the resulting high mortality rate is, in part, explained by a highly immune-suppressive TME [10]. The specific mechanisms underlying the unusually robust immunosuppressive nature of PDAC tumors and the secreted factors that comprise this uniquely inhibitory TME are poorly understood. Recent evidence from multiple investigators suggests phosphatidylserine (PS), a negatively charged phospholipid that is highly expressed in PDAC and other cancer cells compared to healthy cells [11], may have a role in regulating the TME [12]. High surface PS leads to immune suppression and the promotion of tumor growth [12], and the level of PS in cancer cells is highly associated with malignancy and metastatic ability [13]. Several groups, including ours, have demonstrated that cancer surface PS can be targeted by PS-directed drugs [14,15,16]. Saposin C (SapC) is a small, stable lysosomal protein whose physiological role is to activate glucocerebrosidase [17] and, in the context of cancer biology, has a higher binding affinity for PS on cell membranes in the acidic environment typical of tumors [18]. Predictably, SapC specifically targets cancer cells that have higher PS exposure on the outer cell membrane compared to healthy cells. Preclinical and early-phase human studies have shown that the lipid nanovesicle-based PS-targeting drug, SapC coupled with dioleoylphosphatidylserine (SapC-DOPS), localizes to PS-expressing tumor cells and tumor blood vessel endothelial cells [19]. SapC-DOPS is internalized into the cell through endocytosis. After fusion with lysosomes, SapC leads to the activation of acid sphingomyelinase and induces ceramide accumulation in the lysosomes. The elevated ceramide levels then initiate a cascade of events, including caspase activation, which ultimately leads to apoptosis [19,20]. SapC-DOPS has demonstrated a robust anti-tumor effect in solid tumors, including pancreatic cancer [14].

Another component of the TME recently discovered to influence the immune environment within the TME is heat shock protein 70 (Hsp70). Although Hsp70 was initially identified as an intracellular chaperone involved in the cellular stress response [21], Hsp70 is increasingly appreciated to have many other functions [22,23], including in cancer promotion [24,25]. Hsp70 secretion is thought to occur via a non-conventional mode involving lysosomal endosomes or by association with membrane rafts and other secretory proteins [26,27,28]. Although post-translational modifications of Hsp70 such as phosphorylation play critical roles in chaperone function [29,30], a role for Hsp70 phosphorylation in its secretion and or extracellular function is largely unknown. Hsp70 has been shown to be overexpressed in various malignancies [31,32] associated with increased cancer cell proliferation [29,31]. Of specific relevance to this study, elevated levels of both membrane-bound and circulating Hsp70 have been documented in patients diagnosed with pancreatic cancer [32]. In relation to a potential role in the TME, we recently published a study revealing that Hsp70, secreted by PDAC cancer cells, promotes M2 MΦ polarization through the induction of Mer receptor tyrosine kinase (MerTK) upregulation [33].

In this study, we sought to explore the molecular underpinnings of cancer cell innate immune cell crosstalk relevant to pancreatic cancers in which, not only a high surface PS cancer cell phenotype, but also increased expression of Hsp70, is observed. We identified a potentially significant mechanism through which PS operates in the context of PDAC. Elevated levels of surface PS on cancer cells appear to facilitate a non-conventional mode of secretion for Hsp70, thereby enhancing previously reported effects [33] of Hsp70 to promote immunosuppressive M2 MΦs monocyte differentiation. Importantly, we demonstrated a therapeutic strategy to prevent this M2 MΦs induction.

## 2. Materials and Methods

### 2.1. Cell Lines

The cell lines studied were primary human astrocytes (ScienCell Research Laboratories, Carlsbad, CA, USA), primary human pancreatic ductal epithelial (HPDE) (CVCL_0P38) cells, human pancreatic cancer cell lines (AsPC-1 (ATCC-CRL-1682), MiaPaCa-2 (ATCC-CRL-1420), cfPac1-Luc3, Rink-1 and KPC-1624), melanoma cell line (A2058) (ATCC-CRL-3601), lung cancer cell line (H1299 (ATCC-CRL-5803), LLC-GFP), glioblastoma cell lines (Gli36, U373 (ATCC HTB-17), U87ΔEGFR, LN229 (ATCC-CRL-2611)), and human leukemia monocytic cell line THP-1(ATCC-TIB202). Gli36 and U87ΔEGFR cells were a gift from Dr. Balveen Kaur to Dr. Qi; the human pancreatic cfPac1-Luc3 cells were a kind gift from Dr. O. Wildner to Dr. Qi; LLC-GFP (CSC-RRO525, Creative Biogene Inc., Shirley, NY, USA) cells were a kind gift from Dr. John C. Morris to Dr Qi. Rink-1 cells were a kind gift from Drs. Murray Korc and Fazlul Sarkar to Dr. Bogdanov; KPC-1624 cells were a kind gift from Dr. Jen Jen Yeh to Dr. Bogdanov.

We used a range of cell lines to investigate the correlation between PS, Hsp70 expression, and M2 polarization. We aimed to assess whether this relationship holds true across different types of cancer cells. Our findings showed that the correlation was indeed consistent across various high- and low-PS cell lines, including those from glioblastoma, melanoma, and PDAC. While these broader observations were important for our initial analysis, we chose to focus on PDAC in subsequent experiments because of the availability of appropriate mouse models for this cancer type, allowing us to explore the underlying mechanisms in vivo.

### 2.2. Cell Culture

J774 MΦ, KPC-1624, AsPC-1, cfPac-1 Luc, MiaPaCa-2, Rink-1, and THP-1 cells were cultured in RPMI with 25 mM HEPES. Human astrocytes were cultured in an astrocyte cell medium (ScienCell Research Laboratories, Carlsbad, CA, USA) supplemented with the provided growth factor supplements, FBS, and antibiotics. HPDE cells were cultured in a keratinocyte cell medium (Sigma-Aldrich, St. Louis, MO, USA) supplemented with the provided growth factor supplements, FBS and antibiotics. All other cell lines were cultured in DMEM, and all media were supplemented with 10% FBS and 1% penicillin/streptomycin. All cells were cultured in a 5% CO_2_ incubator at 37 °C. Cells were routinely tested for mycoplasma contamination. No cross-contamination was observed in the cell lines, as determined by cellular morphology and growth parameters.

### 2.3. Generation of Exosome/Microparticle-Free Conditioned Media (EMD-CM) from Cancer Cell Lines

Cancer cell lines were grown in their respective media until 70% confluency in 10 cm Corning tissue culture plates (ThermoFisher, Waltham, MA, USA). Then, the media were removed, and the cells were washed twice with serum-free media to remove remnants of serum and dead cells and replenished with serum-free media. After 24 h, conditioned media were collected, centrifuged at 10,000× *g* to remove cellular debris, and followed by ultracentrifugation at 100,000× *g* to remove extracellular exosomes and microparticles (EMD-CM).

### 2.4. THP-1 Differentiation Assay

THP-1 cells (2 × 10^5^) were cultured in 1 mL of EMD-CM adjusted to 100 µg of total cellular protein derived from the specified cancer cell lines. Control THP-1 cells were maintained in DMEM. Following a 24 h incubation period, both control and EMD-CM-treated cells were centrifuged and subsequently incubated with CD14-PE conjugated antibody (eBioscience, San Diego, CA, USA) and PI (ThermoFisher, Waltham, MA, USA) in 100 µL of FACS buffer for 30 min on ice. The cells were then washed with flow cytometry buffer (phosphate-buffered saline (PBS) with 2% FBS), and CD14 expression was evaluated using a BD Fortessa X-20 flow cytometer (BD Biosciences, Cambridge, UK). In experiments designed to assess the role of exosomes and microparticles in THP-1 differentiation, THP-1 cells were cultured for 24 h in unfractionated CM, EMD-CM, or with the exosome/microparticle fraction of the CM. Differentiation was assessed via flow cytometric quantification of CD14 expression as previously described.

### 2.5. Quantification of Hsp70 in Cancer Cells Conditioned Media by ELISA

The concentration of Hsp70 in the EMD-CM was measured using an Hsp70 ELISA Kit (ThermoFisher, Waltham, MA, USA) in accordance with the manufacturer’s protocol.

### 2.6. Preparation of Phospholipid Lipid Vesicles and Treatment of THP-1 Cells

Phospholipid vesicle (PL LV) stock solutions were prepared at a concentration of 1 mM. To prepare the stock, 32 µL of dioleylphosphatidylglycerol (DOPG, Avanti Polar Lipids, Alabaster, AL, USA) (25 mg/mL), 28.8 µL of dioleoylphosphatidic acid (DOPA, Avanti Polar Lipids, Alabaster, AL, USA) (25 mg/mL), 32.5 µL of dioleoylphosphatidylserine (DOPS, Avanti Polar Lipids, Alabaster, AL, USA) (25 mg/mL), 31.5 µL of distearoylphosphatidylcholine (DSPC, Avanti Polar Lipids, Alabaster, AL, USA) (25 mg/mL), or 32 µL of distearoylphosphoglycerol (DSPG, Avanti Polar Lipids, Alabaster, AL, USA) (25 mg/mL) were added into separate glass tubes. The final volume was brought up to 1 mL with chloroform. For the preparation of PL LVs at 50 µM and 100 µM final concentrations, 50 µL and 100 µL of the 1 mM stock solution, respectively, were transferred into glass vials. The organic solvent (chloroform) was removed using nitrogen gas, and a dry lipid film was formed. Following film formation, 1 mL of PBS was added to each vial, and the mixture was bath sonicated in an ice-water bath for 30 min to generate PL LVs. THP-1 cells were treated for 24 h with the indicated cancer cell EMD-conditioned medium (EMD-CM) in the presence of 50 µM to 100 µM of the designated PL LVs.

### 2.7. Preparation of Saposin C-Coupled 1,2-Dioleoyl-sn-glycero-3-phospho-rac-(1-glycerol) (SapC-DOPG) Lipid Vesicles (LVs)

A quantity of 6.4 µL DOPG (25 mg/mL) was dried under a stream of N_2_ gas and mixed with 0.4 mg recombinant SapC protein in 20 µL of citrate-phosphate buffer (pH 5.0). A quantity of 1 mL of PBS was added to the mixture. The Saposin C-coupled DOPG (SapC-DOPG) mixture was then gently sonicated in ice water for 30 min. To fluorescently label the SapC-DOPG lipid vesicles (LVs), we added 30 μL CellVue Maroon (CVM; 1 mM stock in ethanol; Molecular Targeting Technologies, West Chester, PA, USA) to DOPG and dried them together before the addition of SapC and 1 mL PBS. After bath sonication, unbound CVM was removed from the nanovesicle suspension by filtration through a Sephadex G25 column (PD-10; Amersham–Pharmacia Biotech, Piscataway, NJ, USA).

### 2.8. Characterization of Lipid Vesicles (LVs)

Particle sizes and zeta potential were measured by a Nano partica SZ-100V2 (Horiba, Irvine, CA, USA) nanoparticle analyzer.

### 2.9. Cell Sorting

The indicated cancer cell lines were stained with annexin V-FITC (Invitrogen, Waltham, MA, USA) and propidium iodide (PI, BD PharMingen, San Jose, CA, USA), according to the manufacturer’s protocol. Briefly, 1 × 10^6^ cells were incubated with annexin V binding buffer (Invitrogen, Waltham, MA, USA) together with PI for 30 min at room temperature. Subsequently, the cells were washed with annexin V buffer and resuspended in this buffer. Cells with low and high annexin V signals were then gated and sorted with a BD FACS Aria II (BD Bioscience, San Jose, CA, USA).

### 2.10. Immunofluorescence Staining

Cells were seeded on gelatin (0.01%)-coated coverslips. After a 5 h incubation, cells were washed twice with PBS. Then, the cells were incubated overnight at 4 °C with anti-Hsp70 antibody (Abcam, Cambridge, UK) followed by anti-rabbit IgG (H+L), F(ab’)2 fragment AlexaFluor 555 (Cell Signaling Technologies, Danvers, MA, USA) for 1 h at room temperature. After washing with PBS, the cells were stained with FITC-conjugated Annexin V (ABP Biosciences, Rockville, MD, USA), and then mounted using Fluoro-gel II with DAPI and analyzed using a BX51 fluorescence microscope (Olympus, Tokyo, Japan).

### 2.11. Western Blotting Analyses

For Western blot analysis, 50 µg of whole cell lysates in RIPA buffer (Sigma-Aldrich, St. Louis, MO, USA) were denatured using sodium dodecyl sulfate (SDS)-loading dye (Bio-Rad Laboratories, Hercules, CA, USA) and subsequently loaded onto 4–15% denaturing polyacrylamide gradient gels (Bio-Rad Laboratories, Hercules, CA, USA). Following electrophoresis, proteins were transferred to nitrocellulose membranes. The membranes were blocked with 5% non-fat dry milk in PBS with 0.1% Tween-20, after which protein-specific antibodies were added, and the membranes were incubated overnight at 4 °C. The blots were washed three times with PBS-Tween-20 and then incubated with HRP-coupled secondary antibodies. After washing with PBS-Tween-20 three times, the blots were developed using SuperSignal West Dura (ThermoFisher, Waltham, MA, USA). The images were developed at Biorad GelDoc XR+ (Bio-Rad Laboratories, Hercules, CA, USA).

### 2.12. Cell Viability Assay

The MTT (3-[4,5-dimethylthiazol-2yl]-2,5-diphenyl-tetrazolium bromide) assay was performed using the manufacturer’s protocol (Roche Applied Science, Mannheim, Germany) to evaluate cell viability. Briefly, cancer cells (5 × 10^3^) were seeded onto 96-well plates and incubated overnight in a 5% CO_2_ incubator at 37 °C. The cells were treated with DOPG or SapC-DOPG LVs for 48 h, and a viability assay was performed.

### 2.13. Subcutaneous Cancer Cell Implantation and DOPG Lipid Vesicle (LVs) Treatment

Lewis lung carcinoma cells expressing green fluorescent protein (LLC-GFP) (1 × 10^5^) were subcutaneously implanted in 6–8-week-old C57BL/6J mice, with five mice per group, comprising both male and female. The treatment group received DOPG nanoparticles in 200 μL of PBS at a dosage of 1.6 mg/kg per mouse, while the control group received 200 μL of PBS per mouse. Treatment commenced for two days post-implantation and was administered three times a week until the conclusion of the study. Tumor growth was monitored daily by measuring tumor volume with Vernier calipers. Tumor volumes were calculated using the formula V = (π/6)LW^2^ (where V is volume, L is length, and W is width). Tumors were harvested once the volume reached 500 mm^3^ for subsequent histological and tumor MΦ analyses.

### 2.14. Generation of Orthotopic PDAC Tumors in Mice

Rink-1 or KPC-1624 cells (2 × 10^6^) were injected into the pancreata of 6–8-week-old C57BL/6J mice (10 mice per group, comprising both male and female). The treatment regimen involved the administration of DOPG liposomes (in 200 μL PBS, DOPG dosage of 1.6 mg/kg per mouse), SapC-DOPG liposomes (in 200 μL PBS, containing SapC at 4 mg/kg and DOPG at 1.6 mg/kg per mouse), or control saline (200 μL saline per mouse) via tail vein injection. Treatments started one day after the implantation and were administered three times a week until the treatment was completed. For the KPC-1624 and Rink-1 models, 5 and 10 mice were used per treatment group, respectively. All animal procedures followed the guidelines set forth by the National Research Council’s “Guide for the Care and Use of Laboratory Animals” and were approved by the University of Cincinnati Institutional Animal Care and Use Committee (IACUC) (Protocol No. 22-10-25-01).

### 2.15. Phospholipids and SapC-DOPG LVs Binding Assays

PL and SapC-DOPG LVs were prepared as described above, except that HEPES buffered saline was used instead of PBS. Cancer cell EMD-CMs were incubated with indicated PL and SapC-DOPG LVs with the indicated concentration for 3 h rotating at RT, then ultracentrifuged at 100,000× *g* for 1 h. Both pellet and supernatant fractions were used for ELISA. Prior to the analysis of the pellets, the pellets were incubated with ice-cold lysis buffer to release the protein.

### 2.16. PK/PD and Tissue Distribution of SapC-DOPG LVs

SapC-DOPG-CVM LVs was generated using DOPG labeled with a CVM. SapC-DOPG-CVM LVs were assembled using 0.13 mg SapC, 0.08 mg DOPG, and 30 µg CVM per mouse and were used to study organ distribution in mice after intravenous injection into orthotopic PDAC tumor-bearing C57BL/6 mice at different time points. A PDAC tumor model was established as mentioned above. After the tumor was established, tumor-bearing C57BL/6 mice were organized into three mice/group for seven different time points: 10 min, 30 min, 1, 2, 4, 24, and 48 h. SapC-DOPG-CVM LVs (0.13 mg SapC, 0.08 mg DOPG, and 30 µg CVM per mouse) were administered through the tail vein. Blood and organs (brain, ovary, prostate, pancreas, liver, lung, intestine, and heart) were collected at the indicated time points. The Spectrum In Vivo Imaging System (PerkinElmer, Hopkinton, MA, USA) was used for imaging with the Cy5 filter ranging from 640 to 680 nm. Non-compartmental analysis was performed using PKanalix software (version 2019R2, Lixoft SAS, Antony, France). Maximum observed plasma concentration (i.e., C_max_), the time taken to reach C_max_ (i.e., T_max_), the time required to reduce the concentration of drug by half (i.e., T_1/2_), and the average time a molecule stays in the body (i.e., MRT) were determined directly from the individual data. The cumulative area under the plasma concentration–time curve (AUC) from time zero to infinity (i.e., AUC_0-inf_) was derived from the clearance and the dose. AUC was evaluated using the trapezoidal rule.

### 2.17. Toxicity Studies of SapC-DOPG LVs

SapC-DOPG LVs were prepared as mentioned above. C57BL/6 mice at six weeks of age were randomly grouped into two groups of six mice each (three males and three females/group). One group was injected via the tail vein with SapC-DOPG LVs (SapC = 34.4 mg/kg and DOPG = 21.4 mg/kg). Human equivalent dose (HED) was calculated based on body surface area using the equations (Equations (1) and (2)) [34] shown below:

*HED*: Human equivalent dose;

*Km*: Correction factor;

NOAEL: No adverse effect levels of the drug from preclinical toxicological studies were observed.

(1) $$HED\left(\frac{mg}{kg}\right)=Animal\;NOAEL\;\left(\frac{mg}{kg}\right)\times \;{\left({Weight}_{animal}[kg]/{Weight}_{human}[kg]\right)}^{\left(1-0.67\right)}$$

(2) $$HED\left(\frac{mg}{kg}\right)=Animal\;does\;\left(\frac{mg}{kg}\right)\times \;\left({Animal\;K}_{m}/{Human\;K}_{m}\right)$$

The NOAEL of SapC-DOPS, which is in Phase II clinical trials, was used as a reference. The injections were administered daily for the first week and then every two days on the following week. Mouse weights were measured daily, and blood was collected from all mice after the second week. The mice were euthanized after 21 days, and their organs were collected for toxicity analyses.

### 2.18. Quantitative Analysis of Fluorescence Microscopy Images

Image J software (version 1.38e, NIH, Bethesda, MD, USA) was used to calculate the area of total cells and colocalization of PS and Hsp70. Ten randomly selected cells were used for calculations. Statistical analysis was performed using GraphPad Prism 6 Software (GraphPad Software Inc., San Diego, CA, USA). The data were analyzed using a Student’s paired *t*-test or ANOVA followed by Bonferroni’s post hoc test.

### 2.19. Hematological Analyses

Blood samples were collected from six mice, comprising three males and three females, utilizing the retro-orbital method. Briefly, a capillary tube was carefully inserted behind each eye into the retrobulbar venous sinus, facilitating blood collection through capillary action into a collection tube. A total of 20 μL of blood was obtained from each mouse, and hematological analysis was conducted using the Hemavet 950 (Drew Scientific, Plantation, FL, USA).

### 2.20. Statistical Analysis

All statistical analyses were conducted using GraphPad Prism 6 software. The data were analyzed using a Student’s paired t-test or ANOVA followed by Bonferroni’s post hoc test. In vitro experiments were performed two to three times. In vivo experiments were conducted at least three times. * *p* < 0.05, ** *p* < 0.01, *** *p* < 0.001, and **** *p* < 0.0001.

## 3. Results

### 3.1. PS^high^ Cancer Cells Promote Monocyte Differentiation and M2 Polarization via Secreted-Hsp70

**Figure 1:**
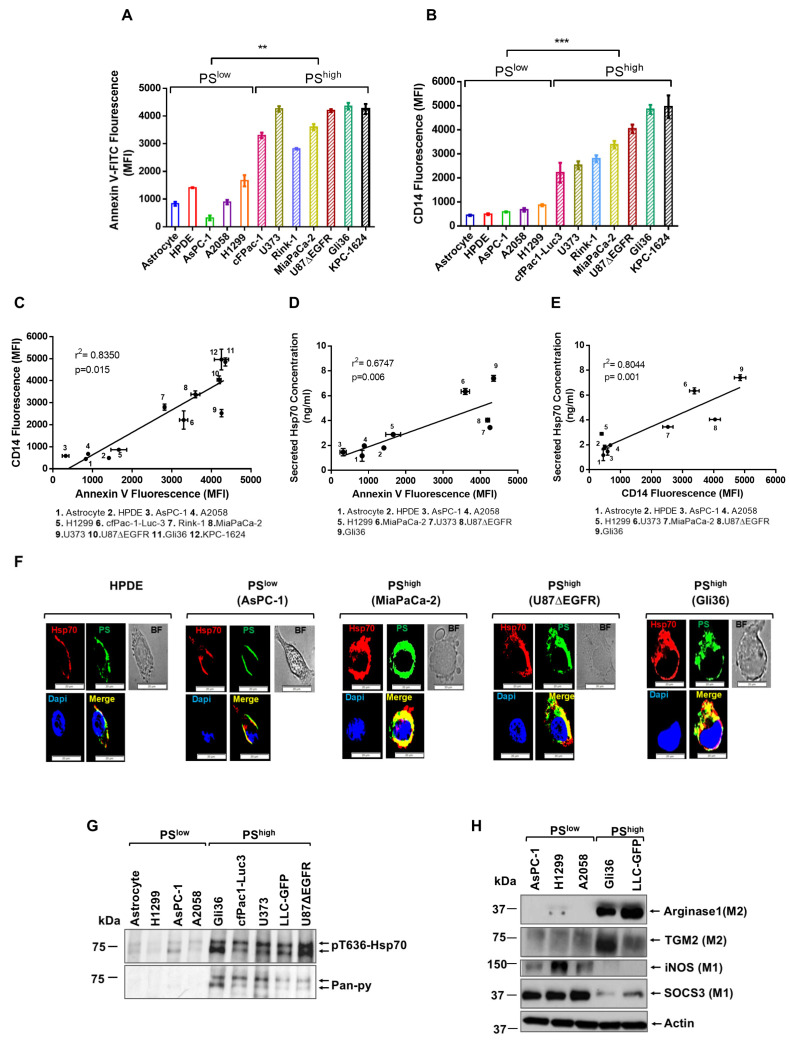
High-PS (PS^high^) cancer cell EMD-CM induces THP-1 differentiation and MΦ M2 polarization. (**A**) Flow cytometric measurement of annexin V on indicated primary cells and cancer cell lines. (**B**) Flow cytometric measurement of CD14 on THP-1 cells cultured for 24 h with EMD-CM from PS^low^ and PS^high^ cancer cells. (**C**) Correlation between surface PS and THP-1 differentiation ability from indicated primary and cancer cell lines, as measured by flow cytometric measurement of annexin V on cancer cell lines and CD14 on EMD-CM-treated THP-1 cells. (**D**) Correlation between surface PS and secreted Hsp70 from indicated primary and cancer cell lines, as measured by flow cytometric measurement of annexin V and ELISA-quantified Hsp70 from the EMD-CM of indicated cells. (**E**) Correlation between THP-1 differentiation ability and secreted Hsp70 from indicated primary and cancer cell lines, as measured by flow cytometric measurement of CD14 on EMD-CM-treated THP-1 cells and ELISA-quantified Hsp70 from the EMD-CM of indicated cells. (**F**) Immunofluorescence microscopic analyses of surface expression of Hsp70 (red), surface PS (green), and their colocalization in HPDE, AsPC-1, MiaPaCa-2, U87∆EGFR, and Gli36 cells. Yellow and blue colors represent Hsp70-PS colocalized area and nuclei, respectively. The bright field image is shown to the right of each panel. (**G**) Western blot analyses of the EMD-CM from indicated PS^low^ and PS^high^ cell lines probed with indicated mAbs. (**H**) Western blot analyses of J774 cell lysates made from cells cultured for 24 h with the EMD-CM from indicated cancer cell lines probed with indicated mAbs. The experiments were repeated at least twice. ** *p* < 0.01, and *** *p* < 0.001. Original western blots are presented in Supplementary Materials File Blots (File S1).

In our previous study, we identified a non-chaperone function of cancer cell-secreted Hsp70. Our results revealed that cancer cell-secreted Hsp70 induces MerTK upregulation and promotes polarization of immunosuppressive M2 macrophages [33]. The promotion of an immunosuppressive TME by Hsp70 released from cancer cells suggests a highly regulated process. Hsp70 is thought to be secreted through a non-classical mechanism involving membrane rafts [35]. Previous studies also showed that PS derived from apoptotic tumor cells correlated with the M2-like phenotype of peritoneal MΦs in vitro [36]. Given that PS is highly expressed on the surface of cancer cells [37,38], correlated with MΦ polarization, and documented to capture Hsp70 [39], we examined the correlation between cancer cell surface PS, the secretion of Hsp70, and MΦ differentiation. Cancer cells were grouped into PS^low^ and PS^high^ lines by annexin V staining (Figure 1A). As expected, EMD-CMs from PS^high^ cells induced robust THP-1 differentiation based on CD14 expression compared to PS^low^ cells such as primary human astrocytes, primary human pancreatic duct epithelial (HPDE) cells, and AsPC-1 cancer cells (Figure 1B). A positive correlation was evident between cancer cell surface PS and CD14 expression induced on the MΦs (r^2^ = 0.8396, *p* = 0.0006) (Figure 1C). Using a Hsp70 ELISA, we assessed whether PS^low^ and PS^high^ cancer cells differed in the amount of secreted Hsp70 and showed that released Hsp70 levels strongly correlated with cell surface PS (Figure 1D; r^2^ = 0.6747, *p* = 0.0066). Moreover, Hsp70 correlated with THP-1 differentiation (Figure 1E; r^2^ = 0.8044, *p* = 0.001).

Since PS^high^ cells secreted more Hsp70, we assessed whether Hsp70 and membrane PS colocalize on the cell surface. We compared the extent of colocalization of PS and Hsp70 in HPDE, PS^low^ AsPC-1 cells, PS^high^ MiaPaCa-2, Gli36, and U87ΔEGFR cells. Colocalization of Hsp70 and PS on the cell membrane was greater in PS^high^ cells (Gli36, U87∆EGFR, and MiaPaCa-2) (Figure 1F and Figure S1) compared to normal HPDE cells and PS^low^ cells (AsPC-1) (Figure 1F and Figure S1). Interestingly, the secreted Hsp70 was phosphorylated at T636 and tyrosine residues and more abundant in PS^high^ cell culture media (Figure 1G). Notably, J774 MΦ cells treated with the EMD-CMs from PS^high^ cells (Gli36 and LLC-GFP) showed marked increases in the M2 polarization markers, arginase 1, and transglutaminase 2 (TGM2), with corresponding decreases in the M1 markers NOS2(iNOS) and SOCS3, compared to PS^low^ cell lines AsPC-1 and H1299 (Figure 1H). These results support a positive correlation between cancer cell surface PS and secreted Hsp70.

**Figure 2:**
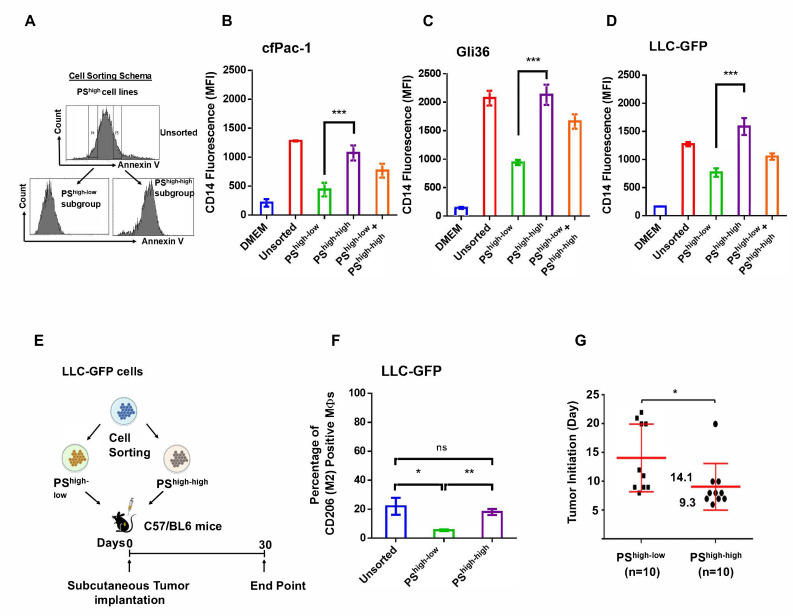
Sorted PS^high-high^ cells from individual cancer cell lines induce strong THP-1 differentiation and M2 MΦ polarization, and initiate tumor faster. (**A**) Scheme showing flow cytometric sorting of cells. Flow cytometric measurement of CD14 on THP-1 cells cultured with DMEM medium, unsorted, sorted PS^high-low^, PS^high-high^ cells, and a mix of both PS^high-low^ and PS^high-high^ cells from cfPac-Luc3 (**B**), Gli36 (**C**), and LLC-GFP (**D**) cell lines. (**E**) Scheme showing subcutaneous implantation in mice with sorted PS^high-low^ or PS^high-high^ LLC-GFP cells. (**F**) CD206 expression on mouse peritoneal MΦs polarization in response to unsorted, PS^high-low^, and PS^high-high^ LLC-GFP cells, measured by flow cytometry. (**G**) Tumor initiation in mice by sorted PS^high-low^ and PS^high-high^ LLC-GFP cells. The experiments were repeated at least twice. ns: not significant, * *p* < 0.05, ** *p* < 0.01, and *** *p* < 0.001.

Recent studies have revealed that tumor cells are highly heterogeneous with distinct cellular subsets and distinct functions [40,41]. We hypothesized the presence of subsets of cells that differ in surface PS and secretion of Hsp70. To test this possibility, PS^high^ cell lines were sorted by flow cytometry after annexin V staining into two subgroups, PS^high-low^ and PS^high-high^ (Figure 2A). Controls were DMEM, unsorted, and a mixture of the two sorted subgroups from individual cancer cell lines (cfPac1-Luc3, Gli36, and LLC-GFP). The PS^high-high^ cells exhibited higher THP-1 differentiation capacity compared to PS^high-low^ cells from cfPac1-Luc3, Gli36, and LLC-GFP (Figure 2B–D). Furthermore, we observed that PS^high-high^ LLC-GFP cells induced stronger M2 polarization when injected into the peritonea of C57BL/6J mice compared to PS^high-low^ LLC-GFP cells (Figure 2E,F). The sorted PS^high-high^ LLC-GFP cells initiated tumors faster than PS^high-low^ cells when injected subcutaneously into C57BL/6J mice (Figure 2G). These findings reveal the correlation between surface PS and CD14 expression and tumor initiation.

### 3.2. DOPG Lipid Vesicles (LVs) Block EMD-CM-Induced Monocyte Differentiation and Increase Survival in Mice

**Figure 3:**
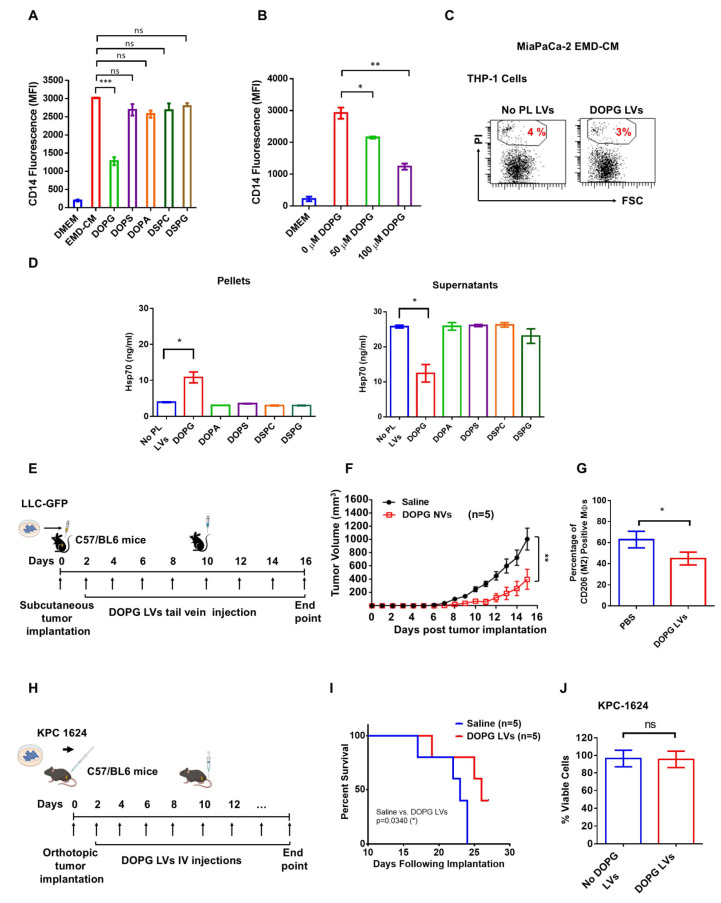
DOPG LVs reduce M2 MΦ, inhibit tumor growth, and increase overall survival in subcutaneous and PDAC orthotopic mouse models. CD14 expression of THP-1 cells incubated with MiaPaCa-2 EMD-CM and indicated LVs at 100 μM (**A**) and DOPG LVs with indicated concentrations (**B**). (**C**) Flow cytometric dot plots showing the percentage of PI positive cells upon incubation of THP-1 cells with MiaPaCa-2 EMD-CM and MiaPaCa-2 EMD-CM together with 100 μM of the DOPG LVs. (**D**) Quantification of Hsp70 by ELISA in pellets (left panel) and supernatant (right panel) obtained after 3 h incubation of MiaPaCa-2 EMD-CM with 100 μM of the indicated PL LVs and ultracentrifugation at 100,000× *g* for 1 h. (**E**) Schematic showing implantation of LLC-GFP cells into mouse flank and DOPG LV treatment regimen and (**F**) tumor volume in mice in response to PBS injection or DOPG LV injection. DOPG concentration was 1.6 mg/kg per mouse. (**G**) Percentage of intra-tumoral MΦs that are CD206-positive (gated on F4/80-positive cells) in mice injected with PBS/DOPG LVs. (**H**) The schematic showing orthotopic implantation of KPC-1624 cells into the mouse pancreas and the DOPG LVs treatment regimen. DOPG concentration was 1.6 mg/kg per mouse. (**I**) The Kaplan–Meier survival curve of the KPC-1624 orthotopic mice treated with saline or DOPG LVs. (**J**) MTT viability analysis of 0 and 100 μM DOPG LVs on KPC-1624 cells. The experiments were repeated at least twice. ns: not significant, * *p* < 0.05, ** *p* < 0.01, and *** *p* < 0.001.

Our previous study demonstrated that Hsp70, secreted by cancer cells, interacts with toll-like receptor 2 (TLR2) and triggers the upregulation of MerTK, thereby stimulating M2 MΦ polarization and contributing to tumor growth [33]. The identification of this novel immunosuppressive pathway suggests innovative therapeutic potential. Hsp70 is known to bind phospholipids (PLs) [39,42]. Thus, we hypothesized that this might serve to inhibit the extracellular bioactivity of phosphorylated Hsp70. Consequently, we analyzed LVs composed of a panel of PLs differing in saturation and head groups for their ability to block THP-1 differentiation induced by cancer cell EMD-CM. MiaPaCa-2 EMD-CM cultured THP-1 cells treated with DOPG LVs showed substantial dose-dependent inhibition of CD14 expression (Figure 3A,B). In contrast, DOPA, DOPS, DSPC, and DSPG LVs elicited only mild reductions in CD14 expression (Figure 3A). Since PI staining of THP-1 cells treated with 100 μM DOPG LVs was comparable to PI staining of THP-1 cells without LV treatment (Figure 3C), the observed effect of DOPG LVs on THP-1 differentiation is unlikely to be attributable to toxicity.

Next, we measured the particle size and zeta potential of LVs to evaluate their physical properties. The results revealed that the average size of DOPG LVs was 258 nm (standard deviation (S.D): 42.1 nm) (Table S1). In contrast, SapC-DOPG LVs were significantly smaller, with an average size of 116 nm (S.D: 40 nm). The smaller size of the SapC-DOPG LVs suggests that SapC may be influencing lipid organization, likely by reducing aggregation or enhancing vesicle formation, leading to smaller, more stable particles. Smaller vesicles are characterized by enhanced cellular uptake, easier penetration of biological barriers, and improved stability. We also evaluated the zeta potential of LVs. The zeta potential of DOPG was −10.1 mV. After coupling with SapC, the zeta potential became less negative, at −7.4 mV. This indicates that SapC leads to reduced electrostatic repulsion between vesicles, suggesting that the coupling of SapC has altered the surface charge of DOPG.

We then tested whether DOPG LVs bind to Hsp70 present in EMD-CM. After incubation of MiaPaCa-2 EMD-CM with PL LVs, we applied ultracentrifugation to separate LVs-bound-Hsp70 in the pellet from its free form in the supernatant. ELISA results demonstrated that Hsp70 bound specifically to DOPG LVs compared to the other PL LVs, as shown by a significant increase of Hsp70 in the DOPG LVs pellet (Figure 3D, left panel) and its reduction in the supernatant (Figure 3D, right panel). These results suggested that DOPG LVs may sequester Hsp70 secreted by cancer cells.

Next, the efficacy of the DOPG LVs was tested in a subcutaneous mouse model (Figure 3E). Intravenously administered DOPG LVs did not alter mouse body weight but substantially reduced tumor growth compared to sham mice (Figure S2 and Figure 3F). Importantly, the reduction in tumor growth by DOPG LVs was associated with a significant reduction in the intra-tumor M2 polarized MΦs (Figure 3G). To support the possibility that the immunosuppressive effect of DOPG is at least in part due to binding to cancer cell-secreted Hsp70 (Figure 3D, left and right panels), we used a syngeneic PDAC orthotopic tumor model to test the efficacy of DOPG LVs. The KPC-1624 cell line was used for in vivo study because of its high surface PS and robust CD14-inducing activity (Figure 1A,B). Briefly, 2 × 10^6^ KPC-1624 cells were implanted into C57BL/6 mice pancreata. The mice were treated with DOPG LVs or saline every other day (Figure 3H). A Kaplan–Meier survival analysis demonstrated that DOPG LVs (26 days of median survival) significantly increased the survival of mice compared to the saline control (23 days of median survival) (Figure 3I). Cell viability analysis showed that 100 µM DOPG LVs showed no killing effect in KPC-1624 cells in vitro (Figure 3J).

### 3.3. SapC-DOPG LVs Block EMD-CM-Induced Monocyte Differentiation and Inhibit Tumor Growth

**Figure 4:**
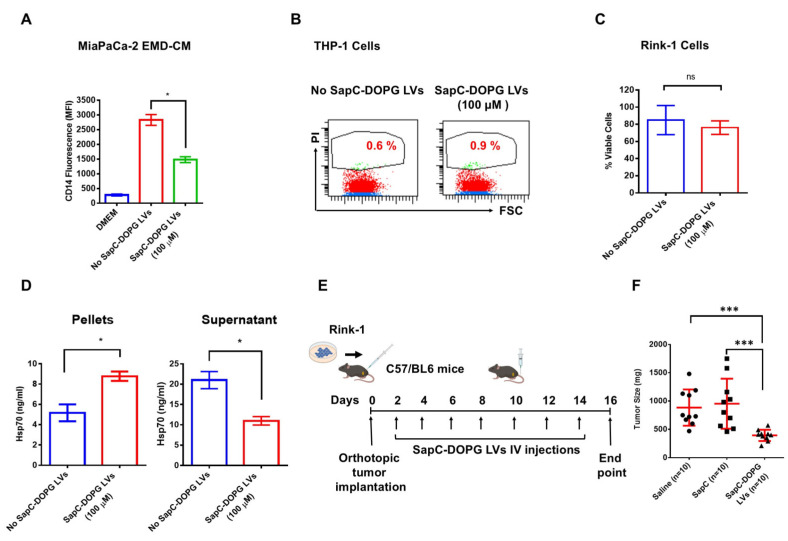
SapC-DOPG LVs block EMD-CM-induced monocyte differentiation and inhibit tumor growth in a PDAC orthotopic mouse model. (**A**) CD14 expression of THP-1 cells incubated MiaPaCa-2 EMD-CM with SapC-DOPG LVs at 0 and 100 μM concentrations. (**B**) Flow cytometric dot plots showing the percent of PI positive cells upon incubation of THP-1 cells with MiaPaCa-2 EMD-CM and MiaPaCa-2 EMD-CM together with 0 and 100 μM of SapC-DOPG LVs. FCS stands for forward scatter. (**C**) MTT viability analysis of 0 and 100 μM SapC-DOPG LVs on Rink-1 PDAC cells. (**D**) Quantification of Hsp70 by ELISA in pellets (left panel) and supernatant (right panel) obtained after 3 h incubation of MiaPaCa-2 EMD-CM with 0 and 100 μM SapC-DOPG LVs and ultracentrifugation at 100,000× *g* for 1 h. (**E**) The schematic showing orthotopic implantation of Rink-1 cells into the mouse pancreata and the SapC-DOPG LVs treatment regimen. SapC-DOPG concentration was SapC = 4 mg/kg and DOPG = 1.6 mg/kg per mouse. (**F**) Orthotopic tumor weights from mice. The experiments were repeated at least twice. ns: not significant, * *p* < 0.05, and *** *p* < 0.001.

Although our toxicity and viability results suggested that DOPG had minimal toxicity to either THP-1 cells or KPC-1624 PDAC cells (Figure 3C,J), studies by others showed that DOPG could induce apoptosis in RAW 264.7 murine macrophage-like cells by air oxidation in vitro [43]. Therefore, we prepared SapC-DOPG LVs. Importantly, the safety of SapC-DOPS (BXQ-350) has already been established in a Phase I clinical trial. First, we tested whether SapC-DOPG LVs block THP-1 differentiation induced by cancer cell EMD-CM. Incubation of MiaPaCa-2 EMD-CM-treated THP-1 cells with 100 µM SapC-DOPG LVs led to substantial inhibition of CD14 expression on THP-1 cells (Figure 4A). Toxicity and cell viability results showed that SapC-DOPG had no toxicity to either THP-1 or Rink-1 PDAC cells (Figure 4B,C). Next, we assessed whether SapC-DOPG LVs bind to cancer cell-secreted Hsp70. ELISA assays demonstrated that SapC-DOPG LVs bound to cancer cell-secreted Hsp70 as indicated by a significant increase of Hsp70 in the ultracentrifuged pellet (Figure 4D, left panel) and its reduction in the supernatant (Figure 4D, right panel). Further, the efficacy of SapC-DOPG LVs was evaluated in a Rink-1 PDAC orthotopic mouse model. Briefly, 2 × 10^6^ Rink-1 cells were orthotopically implanted into the pancreate of C57BL/6 mice. The mice were treated with SapC-DOPG LVs, saline, or SapC every other day for 16 days (Figure 4E) and euthanized, and the tumors were dissected and weighed. Results showed that orthotopic PDAC tumor growth was reduced by about 60% in SapC-DOPG LV-treated mice compared to saline or SapC-treated mice (Figure 4F).

### 3.4. SapC-DOPG LVs Target Pancreatic Tumors Without Body Weight Loss or Organ Toxicity in Mice

**Figure 5:**
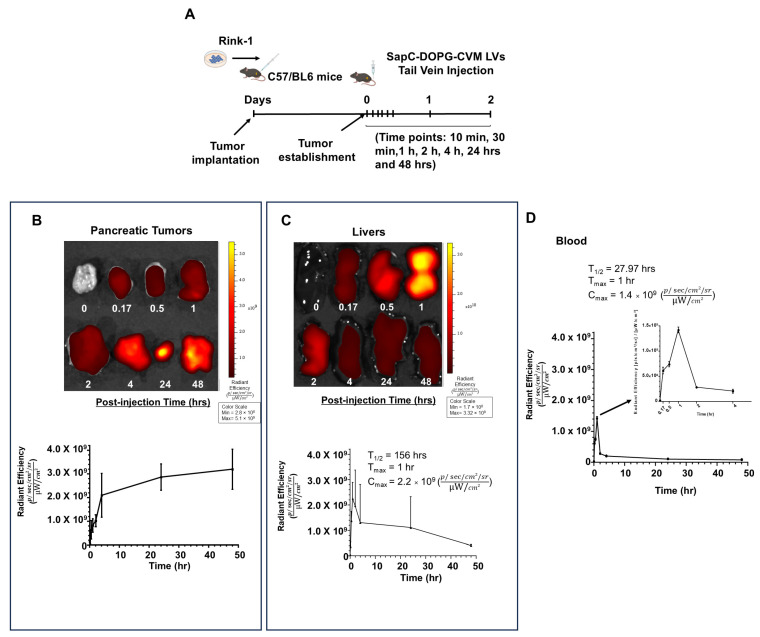
SapC-DOPG LVs target pancreatic tumors. (**A**) Schematic showing orthotopic implantation of Rink-1 cells into mouse pancreas and SapC-DOPG-CVM LV treatment regimen (SapC 6.4 = mg/kg, DOPG = 4 mg/kg, CVM = 0.3 mg/kg per mouse.) (**B**) Fluorescent images of SapC-DOPG-CVM from PDAC tumors at the indicated time points between 0 and 48 h (top panel). The signal of SapC-DOPG-CVM from PDAC tumors at the indicated time points between 0 and 48 h (bottom panel). The signal was normalized based on the area. (**C**) Fluorescent images of SapC-DOPG-CVM from mouse livers at the indicated time points between 0 and 48 h (top panel). The signal of SapC-DOPG-CVM from mouse livers at the indicated time points between 0 and 48 h (bottom panel). The signal was normalized based on the area. (**D**) The signal of SapC-DOPG-CVM from the blood of mice at the indicated time points between 0 and 48 h. The signal was normalized based on the area. The experiments were repeated at least twice.

SapC demonstrates a greater binding affinity for phosphatidylserine (PS) on cell membranes in the acidic environment typical of tumors [18]. It has been shown that SapC-DOPS localizes to PS-expressing tumor cells and tumor blood vessel endothelial cells [18,19]. To test whether Sap-DOPG LVs specifically target tumors in PDAC orthotopic mice, 2 × 10^6^ Rink-1 cells were implanted orthotopically into C57BL/6 mice. After the tumor was established, fluorescently labeled SapC-DOPG-CVM LVs were administered through the tail vein. The blood and body organs were collected at the indicated time points (Figure 5A). Fluorescent intensity of blood and organs was measured and quantified using the Spectrum in vivo Imaging System. Pancreatic tumors showed a gradual increase in CVM signal from SapC-DOPG-CVM LVs, which reached a maximum by 24–48 h, suggesting specific targeting to the tumor (Figure 5B, top and bottom panels). The pharmacokinetic profile of SapC-DOPG-CVM-LVs indicated that the peak concentration of SapC-DOPG-CVM-LVs in the tumor was 3.08 × 10^9^ (C_max_) after 40 h (T_max_) (Table 1). An initial peak of CVM signal intensity in the liver 1 h after SapC-DOPG-CVM LV injection was observed (Figure 5C top and bottom panels). This was diminished and reached the background level by 48 h (Figure 5C, top and bottom panels). A sharp peak of CVM signals in blood was detected within 1 h after injection, followed by a rapid decline within 4 h, and reaching non-detectable levels by 48 h (Figure 5D).

We also examined other organs like the lung, brain, bladder, ovary, kidneys, and heart. Background levels of the CVM signal were recorded from SapC-DOPG-CVM LVs (Figure S3A–F).

**Figure 6:**
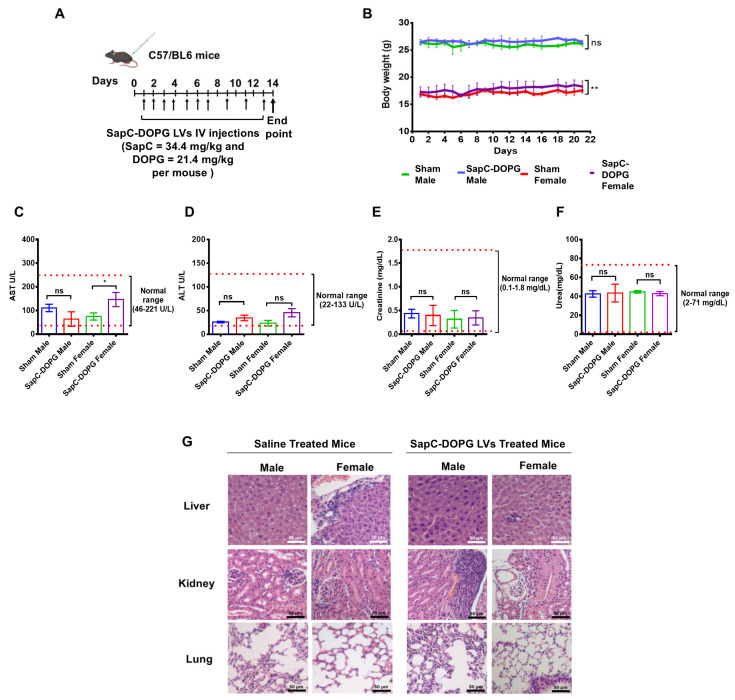
SapC-DOPG LVs have no significant adverse effect on the overall body weight and organs of the mice. (**A**) Schematic showing SapC-DOPG LV treatment regimen. (**B**) Body weights of sham and SapC-DOPG LV-treated male and female mice. ALT (**C**) and AST (**D**) enzymatic analysis from the blood plasma of sham and SapC-DOPG LV-treated mice. Creatinine (**E**) and urea (**F**) analysis from the blood plasma of sham and SapC-DOPG LV-treated mice. (**G**) H&E staining of the liver (top panel), kidney (middle panel), and lung (bottom panel) tissues from sham and SapC-DOPG LV-treated mice. The experiments were repeated at least twice. ns: not significant, * *p* < 0.05, and ** *p* < 0.01.

We further tested whether SapC-DOPG LVs have adverse effects on organs and body weight. Briefly, 6-week-old C57BL/6 mice were randomly divided into two groups of six (three males and three females per group). One group was injected with SapC-DOPG LVs via the tail vein (SapC = 34.4 mg/kg and DOPG = 21.4 mg/kg per mouse) (Figure 6A). Mean body weights of both sham and SapC-DOPG LV-treated mice were comparable (Figure 6B). These results indicate that SapC-DOPG LVs do not have an adverse effect on overall body weight. Next, hematological analyses were conducted using a Hemavet 950. SapC-DOPG LVs did not induce significant changes in the levels of WBC, RBC, HGB, HCT, MCV, MCH, MCHC, or PLT (Table S2). Next, we determined whether SapC-DOPG LVs cause functional damage to the liver and/or kidneys of mice. For liver function, we compared the levels of aspartate aminotransferase (AST) and alanine transaminase (ALT) activity in the plasma of mice injected with saline or SapC-DOPG LVs. Even though there was a slight change (*p* = 0.045) in AST activity between SapC-DOPG female and Sham female, the enzyme activity was still within the normal range (Figure 6C). Further, ALT activity in the plasma of mice revealed that SapC-DOPG LVs showed no significant changes (Figure 6D). Kidney function assessed by creatinine and urea levels showed no significant changes in either the mice treated with SapC-DOPG LVs or the saline group, regardless of sex, as presented in Figure 6E,F. Moreover, histopathology results revealed no significant tissue damage in the organs of mice treated with SapC-DOPG LVs or saline, as assessed by a pathologist (Figure 6G). The livers from both groups showed no sign of fatty change, sinus congestion, hemorrhage, necrotic foci, neoplastic change, microgranulomas, or Kupffer cell swelling (Figure 6G, top panel). Similarly, the kidneys of the control groups displayed no tubular necrosis, widened sinusoids, or neoplastic changes (Figure 6G, middle panel). No evidence of hemorrhage, granuloma, or neoplastic changes was observed in the lungs (Figure 6G bottom panel).

Together, these data indicate that in a mouse model, SapC-DOPG LVs specifically target pancreatic tumors without significant toxicity.

## 4. Discussion

In this study, we show that cancer cells with elevated PS on the cell surface secrete a phosphorylated Hsp70 (pT636-Hsp70) that is soluble and not associated with exosomes or microparticles. Moreover, reinforcing our recent study [33], EMD-CM harvested from cancer cells with elevated surface PS induced robust monocyte differentiation and M2 MΦ polarization. Consistent with a positive correlation between cancer cell surface PS, secreted Hsp70, and CD14 upregulation in THP-1 cells, subpopulations of cancer cells with higher cell surface PS sorted from their original more heterogeneous original lines possessed higher MΦ differentiation and earlier tumor initiation ability. Further, we showed that DOPG LVs are non-toxic to normal cells, bind to cancer-secreted Hsp70, increase the survival of KPC 1624 orthotopic mice model, and reduce the tumor volume and M2 MΦs in LLC-GFP mouse subcutaneous tumors.

Herein, we found larger areas of surface colocalization of Hsp70 and PS in PS^high^ cancer cell lines compared to PS^low^ cancer cells, indicating a PS-associated externalization of Hsp70 in cancer cells. Furthermore, we found a striking correlation between cancer cell surface PS, secreted Hsp70, and MΦ differentiation. Interestingly, cancer cells are known to express higher levels of surface PS compared to normal cells; the higher intracellular calcium in cancer cells reduces flippase activity, leading to higher PS on the cell surface [11]. We have previously found that different types of cancers and even cells within individual cancer types vary in surface PS exposure [11]. As Hsp70 lacks a conventional secretory signal, the mechanism of secretion involves a non-classical pathway via membrane rafts by binding to other proteins. The current study strongly suggests that PS exerts an important role in facilitating non-classical Hsp70 secretion. Consistent with these findings, the endolysosomal route has been described as a release mechanism for membrane-bound, exosomal, and soluble forms of Hsp70 [35].

Our results may reveal an important regulatory mechanism that underlies the cancer-promoting actions of Hsp70. Specifically, we found a positive correlation between cancer cell PS exposure and the quantity of secreted M2 markers, such as arginase 1 and TGM2. Arginase 1 is a key marker for M2 polarization in cancers, significantly contributing to the immunosuppressive TME by depleting L-arginine [44], while TGM2 plays a supporting role in M2 polarization by facilitating the extracellular matrix remodeling and promoting cell adhesion, thereby further enhancing tumor progression [45]. In particular, we demonstrated that cancer subcellular populations with the higher PS cellular fraction possess higher MΦ differentiation ability and faster tumor initiation ability. This suggests that higher surface PS cells in individual cancer types could be targeted to inhibit cancer immunosuppression.

SapC-DOPS is a PS-targeting drug. In preclinic models, it has been shown that SapC-DOPS targets a variety of primary and metastatic tumors including pancreatic cancer [19], glioblastoma multiforme (GBM) [46], and lung cancer [47]. We have shown that SapC-DOPS has an additive response when used in combination with gemcitabine or gemcitabine-abraxane in PDAC models [14]. Also, SapC-DOPS has been shown to cross the blood–brain tumor barrier (BBTB) and has a strong synergistic effect in combination with temozolomide in the glioblastoma model [46]. SapC-DOPS is non-toxic to normal cells or tumor-free organs. BXQ-350 (SapC-DOPS) has completed a Phase I clinical trial. It was well tolerated in humans without any dose-limiting toxicity in the Phase I safety trials in brain tumors in both children and adults [48,49] and has now progressed to a Phase Ib/II clinical trial for newly diagnosed metastatic colorectal carcinoma [50].

It is known that Hsp70 interacts with negatively charged phospholipids in the cell membrane, and this serves to fold nascent Hsp70 proteins or prevent aggregation of membrane-bound proteins. In this study, in vitro binding assay results revealed that negatively charged DOPG has an affinity for positively charged Hsp70. The electrostatic attraction between the positive charges on Hsp70 and the negative charges on DOPG helps to mediate the binding of Hsp70 to DOPG. DOPG LVs are also superior to DOPS LVs in reducing M2 MΦ polarization in THP-1 cells (Figure 3A). Therefore, DOPG LVs were used in vivo efficacy studies. DOPG has demonstrated therapeutic potential. However, concerns regarding its toxicity in certain contexts have been raised in the literature [43]. SapC exhibits a higher binding affinity for phosphatidylserine on cell membranes within the acidic environment of tumors [18]. To enhance the targeted delivery of DOPG to the tumor microenvironment, ensuring that the treatment is more selective to the PDAC cells while minimizing off-target effects and potential toxicity, we coupled SapC to DOPG (SapC-DOPG). Indeed, our results showed that SapC-DOPG specifically targeted the pancreatic tumor and significantly reduced the size of tumor in a Rink-1 orthotopic mouse model. Moreover, SapC-DOPG demonstrated safety in a mouse model, with no observed adverse effects, reinforcing its potential as a therapeutic agent. Additionally, one of the analogs of SapC-DOPG, SapC-DOPS, is already undergoing Phase II clinical trials, and its safety profile has been approved in patients, further supporting the viability and safety of using SapC in combination with phospholipids for targeted therapeutic delivery.

Unlike for SapC-DOPS, we do not have data indicating the potential synergistic or additive effects of SapC-DOPG when combined with existing chemotherapeutic or immunotherapeutic regimens. As SapC-DOPG affects immune surveillance functions, therapies with mechanisms distinct from those primarily affecting immune pathways, such as gemcitabine that directly impair DNA synthesis, are likely to be at least additive and potentially synergistic. Further investigation is required in future studies to clarify this aspect.

Although our study has some limitations, none significantly weaken the overall credibility of the primary findings. First, the role of PS for the secretion of Hsp70 is only partially defined. The inhibition of M2 MΦ polarization in PDAC cancer cells by sequestering Hsp70 via DOPG LVs was largely correlative. While our results demonstrated a positive correlation between cancer cell surface PS and secreted Hsp70, as well as a physical interaction between PS and Hsp70 observed through fluorescence imaging, the specific binding must be further elucidated using the microscale thermophoresis technique (MST). Second, the precise mechanism through which Hsp70 induces the M2 MΦ polarization and the role of different phosphorylated forms are not completely dissected. Third, we do not suggest that Hsp70 is the only and/or the most potent secreted factor in the PDAC TME. In our recent study [11], we show that Hsp70, with its identity confirmed by LC-MS, is preferentially secreted by PDAC cancer cells. However, immuno-neutralizing monoclonal antibodies to Hsp70 resulted in at most a 50% reduction in CD14 expression in THP-1 monocytes. Consistent with these prior results, several studies have indicated that soluble programmed death ligand 1 (sPD-L1), which is a protein secreted by cancer cells and is detectable in the TME, may adversely modulate T-cell functionality. In patients diagnosed with gastric cancer [51] and non-small cell lung cancer [52], elevated serum levels of sPD-L1 have been associated with enhanced metastasis and an increased risk of mortality. Also, recent studies revealed the effect of cancer cell-secreted microRNAs (miRNAs) on immune cell function [53,54]. Fourth, although we observed a positive correlation between PS exposure and secreted Hsp70 in PDAC cells, the specific contribution of PS exposure to the secretion of Hsp70 was not directly investigated. Moreover, how these two phenomena regulate the immunosuppressive landscape of the PDAC tumor microenvironment remains speculative, and future research efforts should aim to clarify this pathway. Fifth, the high-high PS population appears similar in CD14 expression to that of the non-sorted population; we speculate this may be due to the threshold effect of PS exposure. Once this threshold is reached, Hsp70 release is triggered with subsequent macrophage activation. While the low-low cells do not reach this threshold, the non-sorted cells do. Finally, most experiments were performed using EMD-CM from cancer cells rather than a purified or semi-purified form of Hsp70; a more definitive approach and one intended for future studies would be to use purified bioactive Hsp70 that could be quantified by a specific ELISA.

## 5. Conclusions

Our studies reveal a new and important function for Hsp70 protein released from cancer cells. Our previous study revealed that cancer-secreted Hsp70 exerts a marked immunosuppressive effect in TME [33] mediated through MerTK and TLR2 [33]. Our current study extends this previously unknown function of Hsp70 by demonstrating (1) the role of PS in Hsp70 secretion, (2) the inhibition of M2 MΦ polarization in PDAC cancer cells by sequestering Hsp70 via DOPG LVs, and (3) the inhibition of the growth of tumors and the sequestration of Hsp70 by these lipid-binding nanovesicles. Indeed, our findings that cancer cell surface PS is associated with induction of M2 MΦ polarization through cancer cell-secreted Hsp70 opens a novel functional role for tumor cell PS in communication with immune cells. Because elevated cell-surface PS is almost a unique feature of tumor cells, PS, together with cancer cell-secreted Hsp70, provides critical targets to block tumor-induced immune suppression.

## Figures and Tables

**Table 1:** Pharmacokinetic/pharmacodynamic (PK/PD) analysis of pancreatic tumors and organs of mice treated with SapC-DOPG.

Group	AUC_INF_	AUC_last_	C_max_	T_max_	T_1/2_	MRT
	Radiant Efficiency [p/s/cm^2^/sr]*h/[µW/cm^2^]	Radiant Efficiency [p/s/cm^2^/sr]*h/[µW/cm^2^]	Average Radiant Efficiency [p/s/cm^2^/sr]/[µW/cm^2^]	h	h	h
Blood (SEM)	8.622 × 10^9^ (7.947 × 10^8^)	6.170 × 10^9^ (3.326 × 10^8^)	1.184 × 10^9^ (2.129 × 10^8^)	0.83 (0.17)	29.2 (6.08)	37.05 (9.01)
Tumor (SEM)	N/A	1.209 × 10^11^ (1.757 × 10^10^)	3.085 × 10^9^ (4.749 × 10^8^)	40 (8)	N/A	N/A
Liver (SEM)	N/A	4.640 × 10^10^ (2.693 × 10^10^)	2.596 × 10^9^ (6.091 × 10^8^)	1.33 (0.33)	N/A	N/A
Kidney (SEM)	N/A	9.312 × 10^9^ (3.097 × 10^9^)	5.821 × 10^8^ (1.301 × 10^8^)	8.83 (7.6)	N/A	N/A
Spleen (SEM)	N/A	1.739 × 10^10^ (2.409 × 10^9^)	5.295 × 10^8^ (8.229 × 10^7^)	32 (8)	N/A	N/A
Brain (SEM)	8.431 × 10^9^ (1.712 × 10^8^)	5.360 × 10^9^ (8.997 × 10^8^)	3.086 × 10^8^ (4.907 × 10^7^)	8.83 (7.6)	38.38 (14.95)	51.7 (23.08)
Heart (SEM)	5.577 × 10^9^ (3.748 × 10^8^)	4.320 × 10^9^ (5.650 × 10^8^)	2.480 × 10^8^ (5.119 × 10^7^)	9.33 (7.33)	29.37 (4.73)	42.09 (3.32)
Lung (SEM)	N/A	2.119 × 10^10^ (8.078 × 10^9^)	1.051 × 10^9^ (1.821 × 10^8^)	1.17 (0.44)	N/A	N/A
Ovary (SEM)	N/A	1.464 × 10^10^ (4.112 × 10^9^)	4.912 × 10^8^ (1.469 × 10^8^)	9.33 (7.33)	N/A	N/A
Intestine (SEM)	2.351 × 10^10^ (8.036 × 10^9^)	1.302 × 10^10^ (1.034 × 10^9^)	7.441 × 10^8^ (2.281 × 10^8^)	2.33 (0.88)	30.78 (8.61)	47.24 (16.14)
Bladder (SEM)	N/A	1.433 × 10^10^ (7.469 × 10^9^)	6.028 × 10^8^ (1.992 × 10^8^)	40 (8)	N/A	N/A

* AUC, C_max_, T_max,_ T1/2 and Mean residence time (MRT) were calculated using the NCA module of PKanalix (PKanalix version 2020R1. Antony, France: Lixoff SAS, 2020). N/A: not available.

## Data Availability

No new datasets were created that are posted.

## Supplementary Materials

The following supporting information can be downloaded at: https://www.mdpi.com/article/10.3390/cancers17071224/s1, Figure S1: Quantification of exposed PS area, HSP70 area, PS and Hsp70 co-localized areas on cancer cells; Figure S2: Body weights of LLC-GFP subcutaneous mouse model following injection of PBS or DOPG LVs; Figure S3: The signal of SapC-DOPG-CVM measured at mice organs; Table S1. Particle size distribution of the synthesized nanoparticles measured by DLS; Table S2. Hematological analysis of mice injected with SapC-DOPG LVs; File S1: Original western blots.
