# Supplementary material for: Targeting Hsp70 Immunosuppressive Signaling Axis with Lipid Nanovesicles: A Novel Approach to Treat Pancreatic Cancer
Source: Cancers (Basel). 2025 Apr 4;17(7):1224. doi: 10.3390/cancers17071224 (PMC11988048; doi:10.3390/cancers17071224)
Supplement: Supplementary file 1 [file cancers-17-01224-s001.zip › Supplementary Files/02132025_Supplemantary Tables_ Table S1-S2.pptx]

## Slide 1
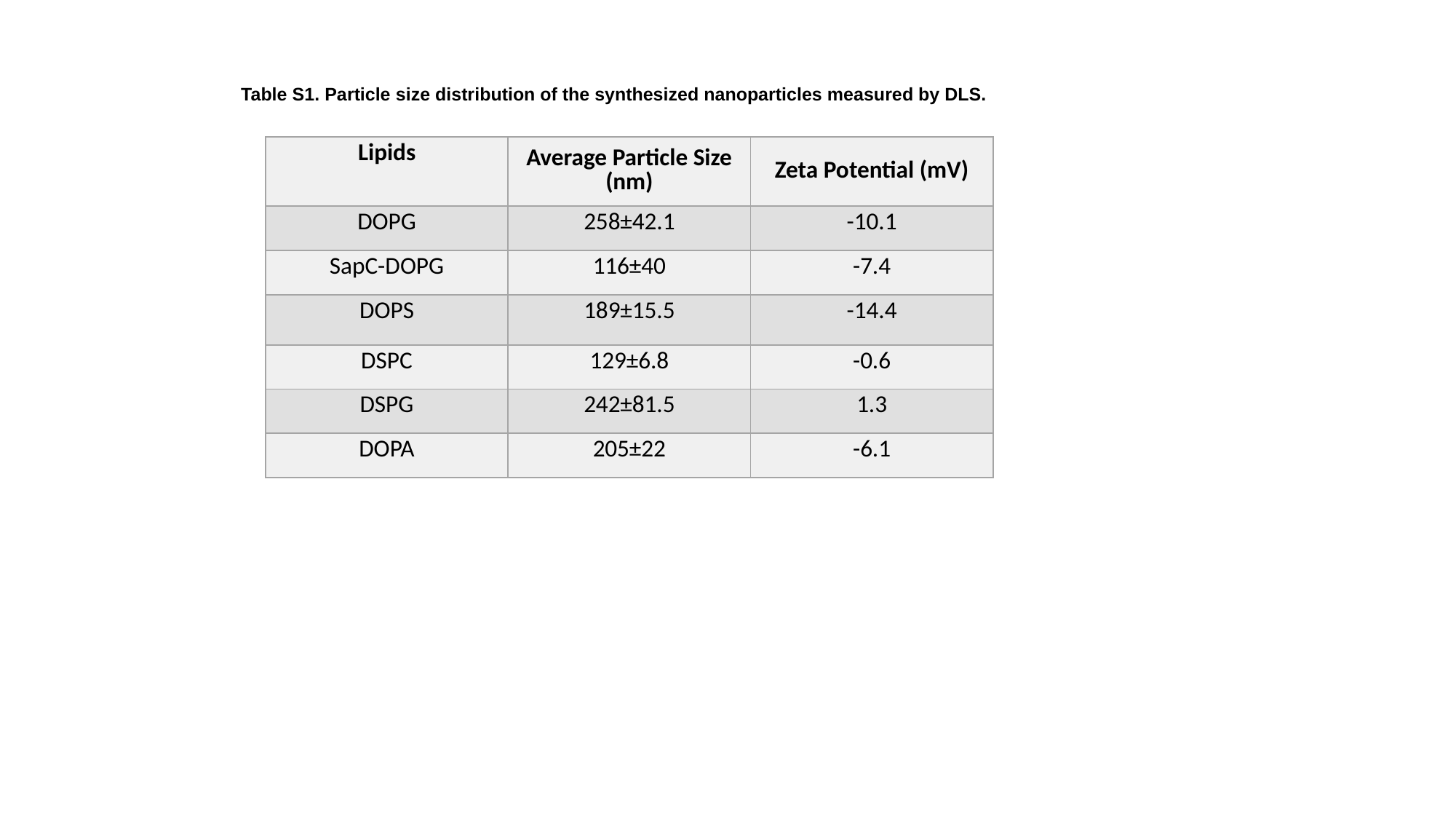

Table S1. Particle size distribution of the synthesized nanoparticles measured by DLS.
| Lipids | Average Particle Size (nm) | Zeta Potential (mV) |
| --- | --- | --- |
| DOPG | 258±42.1 | -10.1 |
| SapC-DOPG | 116±40 | -7.4 |
| DOPS | 189±15.5 | -14.4 |
| DSPC | 129±6.8 | -0.6 |
| DSPG | 242±81.5 | 1.3 |
| DOPA | 205±22 | -6.1 |

## Slide 2
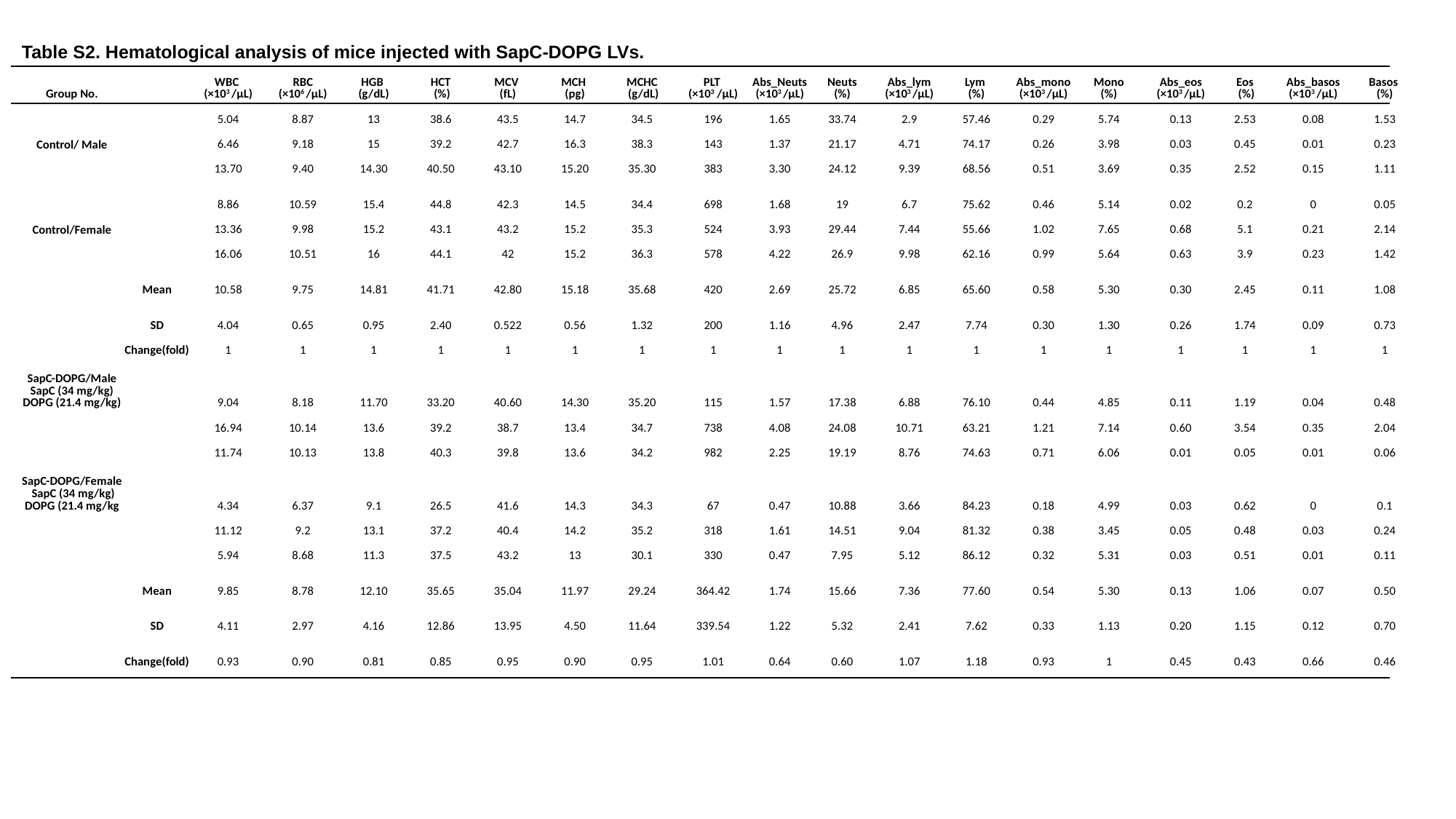

Table S2. Hematological analysis of mice injected with SapC-DOPG LVs.
| Group No. | | WBC (×103 /µL) | RBC (×106 /µL) | HGB (g/dL) | HCT (%) | MCV (fL) | MCH (pg) | MCHC (g/dL) | PLT (×103 /μL) | Abs\_Neuts (×103 /µL) | Neuts (%) | Abs\_lym (×103 /µL) | Lym (%) | Abs\_mono (×103 /µL) | Mono (%) | Abs\_eos (×103 /µL) | Eos (%) | Abs\_basos (×103 /µL) | Basos (%) |
| --- | --- | --- | --- | --- | --- | --- | --- | --- | --- | --- | --- | --- | --- | --- | --- | --- | --- | --- | --- |
| Control/ Male | | 5.04 | 8.87 | 13 | 38.6 | 43.5 | 14.7 | 34.5 | 196 | 1.65 | 33.74 | 2.9 | 57.46 | 0.29 | 5.74 | 0.13 | 2.53 | 0.08 | 1.53 |
| | | 6.46 | 9.18 | 15 | 39.2 | 42.7 | 16.3 | 38.3 | 143 | 1.37 | 21.17 | 4.71 | 74.17 | 0.26 | 3.98 | 0.03 | 0.45 | 0.01 | 0.23 |
| | | 13.70 | 9.40 | 14.30 | 40.50 | 43.10 | 15.20 | 35.30 | 383 | 3.30 | 24.12 | 9.39 | 68.56 | 0.51 | 3.69 | 0.35 | 2.52 | 0.15 | 1.11 |
| Control/Female | | 8.86 | 10.59 | 15.4 | 44.8 | 42.3 | 14.5 | 34.4 | 698 | 1.68 | 19 | 6.7 | 75.62 | 0.46 | 5.14 | 0.02 | 0.2 | 0 | 0.05 |
| | | 13.36 | 9.98 | 15.2 | 43.1 | 43.2 | 15.2 | 35.3 | 524 | 3.93 | 29.44 | 7.44 | 55.66 | 1.02 | 7.65 | 0.68 | 5.1 | 0.21 | 2.14 |
| | | 16.06 | 10.51 | 16 | 44.1 | 42 | 15.2 | 36.3 | 578 | 4.22 | 26.9 | 9.98 | 62.16 | 0.99 | 5.64 | 0.63 | 3.9 | 0.23 | 1.42 |
| | Mean | 10.58 | 9.75 | 14.81 | 41.71 | 42.80 | 15.18 | 35.68 | 420 | 2.69 | 25.72 | 6.85 | 65.60 | 0.58 | 5.30 | 0.30 | 2.45 | 0.11 | 1.08 |
| | SD | 4.04 | 0.65 | 0.95 | 2.40 | 0.522 | 0.56 | 1.32 | 200 | 1.16 | 4.96 | 2.47 | 7.74 | 0.30 | 1.30 | 0.26 | 1.74 | 0.09 | 0.73 |
| | Change(fold) | 1 | 1 | 1 | 1 | 1 | 1 | 1 | 1 | 1 | 1 | 1 | 1 | 1 | 1 | 1 | 1 | 1 | 1 |
| SapC-DOPG/Male SapC (34 mg/kg) DOPG (21.4 mg/kg) | | 9.04 | 8.18 | 11.70 | 33.20 | 40.60 | 14.30 | 35.20 | 115 | 1.57 | 17.38 | 6.88 | 76.10 | 0.44 | 4.85 | 0.11 | 1.19 | 0.04 | 0.48 |
| | | 16.94 | 10.14 | 13.6 | 39.2 | 38.7 | 13.4 | 34.7 | 738 | 4.08 | 24.08 | 10.71 | 63.21 | 1.21 | 7.14 | 0.60 | 3.54 | 0.35 | 2.04 |
| | | 11.74 | 10.13 | 13.8 | 40.3 | 39.8 | 13.6 | 34.2 | 982 | 2.25 | 19.19 | 8.76 | 74.63 | 0.71 | 6.06 | 0.01 | 0.05 | 0.01 | 0.06 |
| SapC-DOPG/Female SapC (34 mg/kg) DOPG (21.4 mg/kg | | 4.34 | 6.37 | 9.1 | 26.5 | 41.6 | 14.3 | 34.3 | 67 | 0.47 | 10.88 | 3.66 | 84.23 | 0.18 | 4.99 | 0.03 | 0.62 | 0 | 0.1 |
| | | 11.12 | 9.2 | 13.1 | 37.2 | 40.4 | 14.2 | 35.2 | 318 | 1.61 | 14.51 | 9.04 | 81.32 | 0.38 | 3.45 | 0.05 | 0.48 | 0.03 | 0.24 |
| | | 5.94 | 8.68 | 11.3 | 37.5 | 43.2 | 13 | 30.1 | 330 | 0.47 | 7.95 | 5.12 | 86.12 | 0.32 | 5.31 | 0.03 | 0.51 | 0.01 | 0.11 |
| | Mean | 9.85 | 8.78 | 12.10 | 35.65 | 35.04 | 11.97 | 29.24 | 364.42 | 1.74 | 15.66 | 7.36 | 77.60 | 0.54 | 5.30 | 0.13 | 1.06 | 0.07 | 0.50 |
| | SD | 4.11 | 2.97 | 4.16 | 12.86 | 13.95 | 4.50 | 11.64 | 339.54 | 1.22 | 5.32 | 2.41 | 7.62 | 0.33 | 1.13 | 0.20 | 1.15 | 0.12 | 0.70 |
| | Change(fold) | 0.93 | 0.90 | 0.81 | 0.85 | 0.95 | 0.90 | 0.95 | 1.01 | 0.64 | 0.60 | 1.07 | 1.18 | 0.93 | 1 | 0.45 | 0.43 | 0.66 | 0.46 |
