# Supplementary material for: Targeting Hsp70 Immunosuppressive Signaling Axis with Lipid Nanovesicles: A Novel Approach to Treat Pancreatic Cancer
Source: Cancers (Basel). 2025 Apr 4;17(7):1224. doi: 10.3390/cancers17071224 (PMC11988048; doi:10.3390/cancers17071224)
Supplement: Supplementary file 1 [file cancers-17-01224-s001.zip › Supplementary Files/02132025-Supplementary Figures.pptx]

## Slide 1
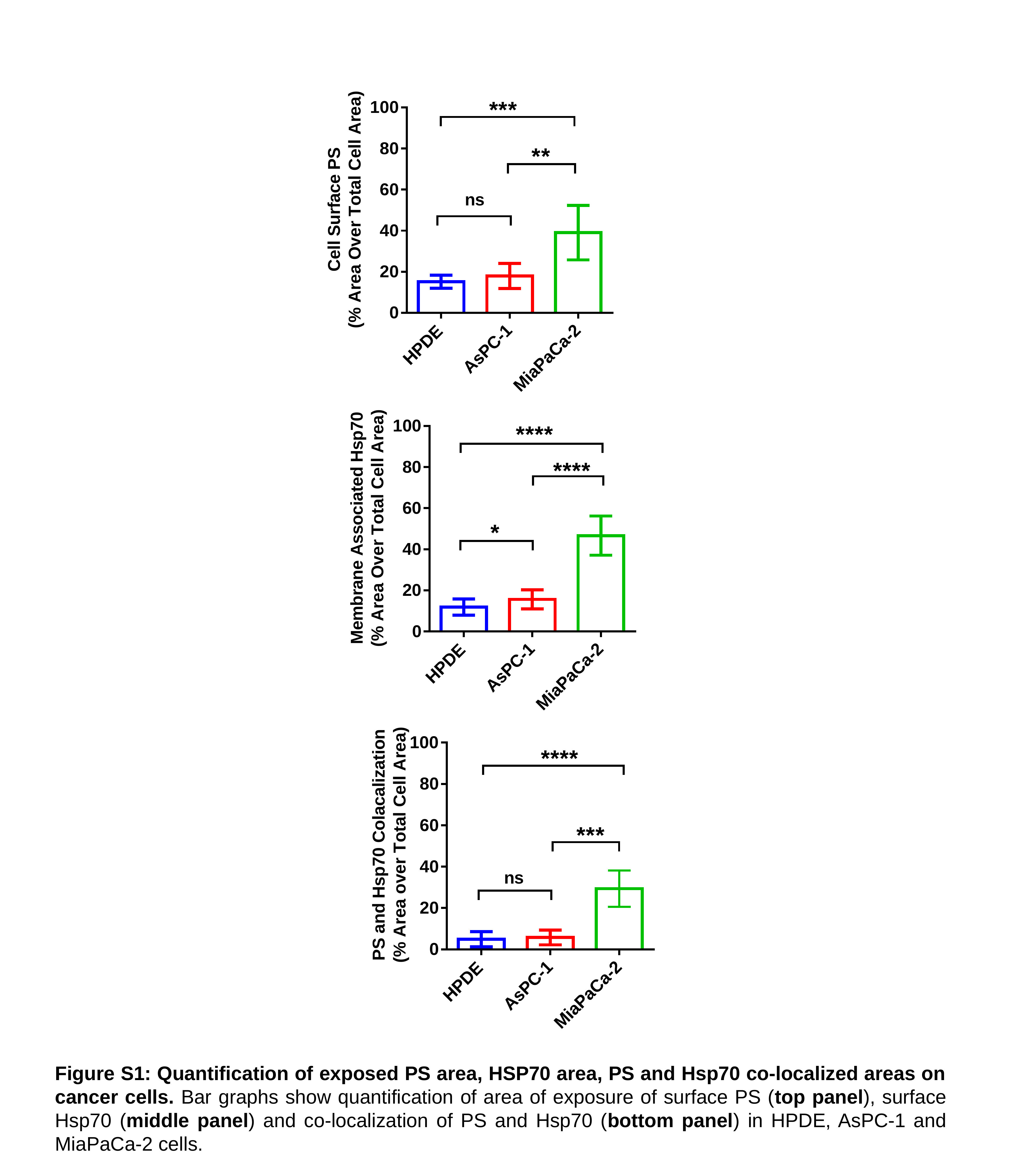

Figure S1: Quantification of exposed PS area, HSP70 area, PS and Hsp70 co-localized areas on cancer cells. Bar graphs show quantification of area of exposure of surface PS (top panel), surface Hsp70 (middle panel) and co-localization of PS and Hsp70 (bottom panel) in HPDE, AsPC-1 and MiaPaCa-2 cells.

## Slide 2
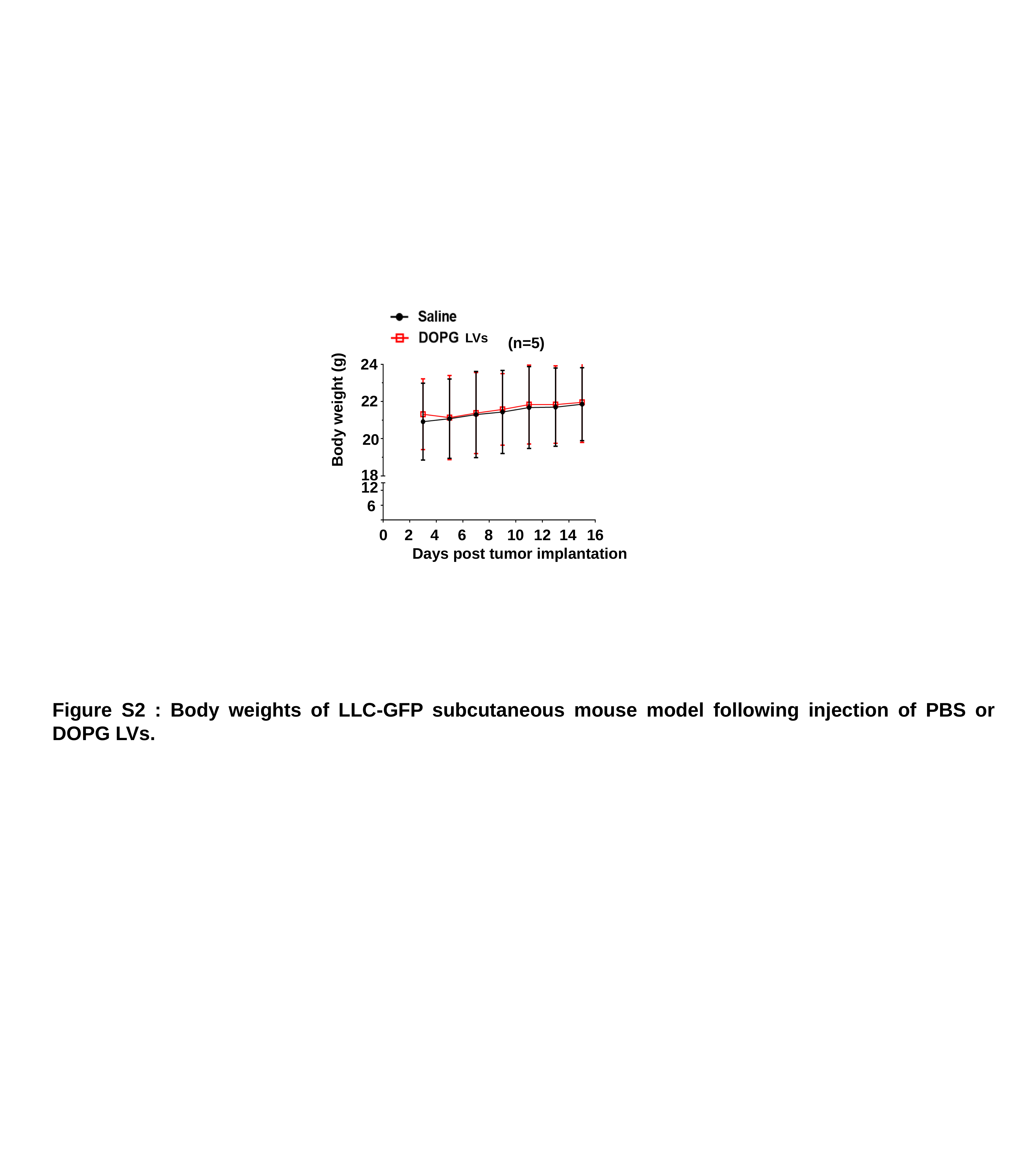

(n=5)
24
22
Body weight (g)
20
18
12
6
0
2
4
6
8
10
12
14
16
Days post tumor implantation
LVs
Figure S2 : Body weights of LLC-GFP subcutaneous mouse model following injection of PBS or DOPG LVs.

## Slide 3
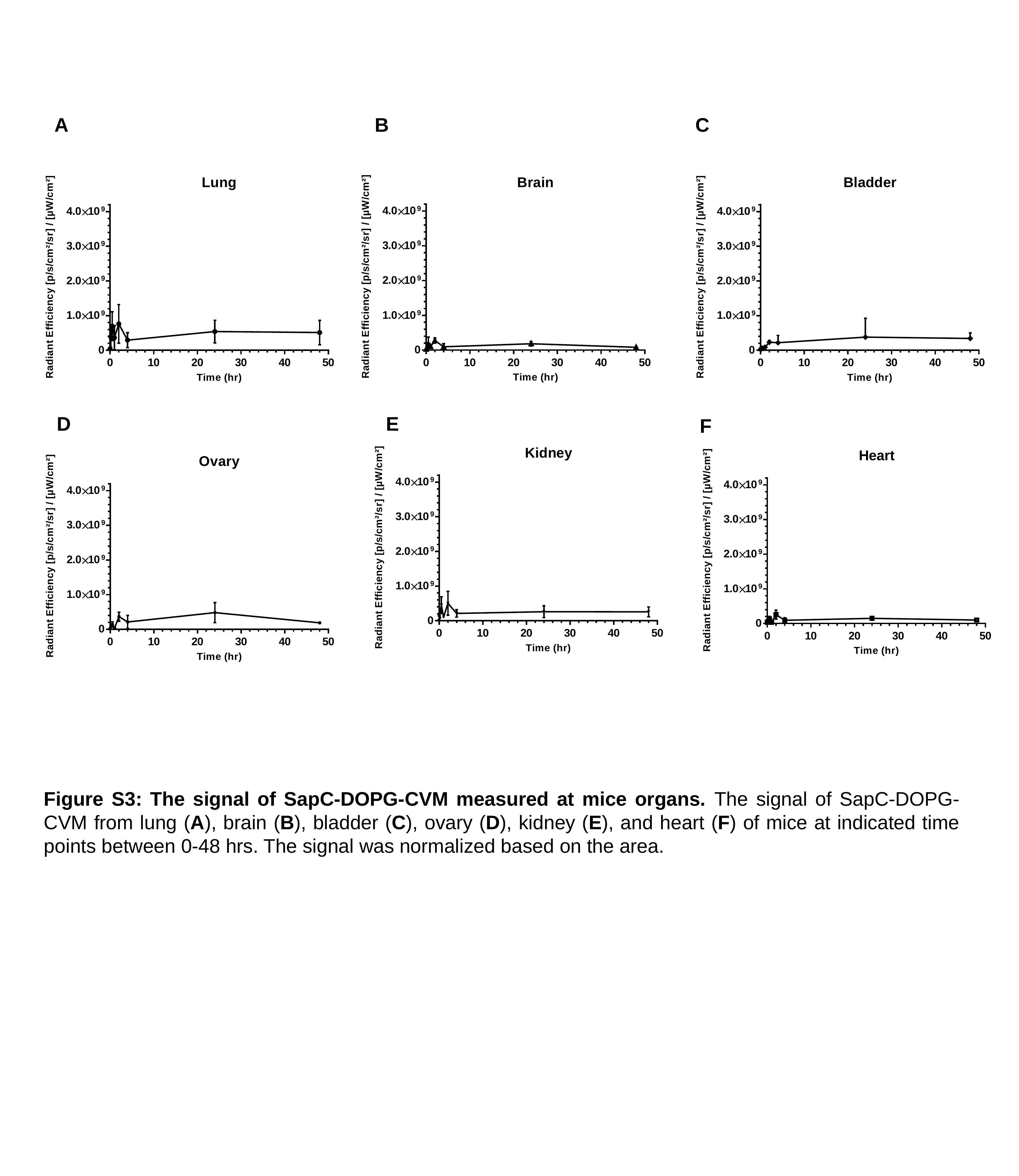

A
B
C
D
E
F
Figure S3: The signal of SapC-DOPG-CVM measured at mice organs. The signal of SapC-DOPG-CVM from lung (A), brain (B), bladder (C), ovary (D), kidney (E), and heart (F) of mice at indicated time points between 0-48 hrs. The signal was normalized based on the area.
