# Supplementary material for: Targeting Hsp70 Immunosuppressive Signaling Axis with Lipid Nanovesicles: A Novel Approach to Treat Pancreatic Cancer
Source: Cancers (Basel). 2025 Apr 4;17(7):1224. doi: 10.3390/cancers17071224 (PMC11988048; doi:10.3390/cancers17071224)
Supplement: Supplementary file 1 [file cancers-17-01224-s001.zip › Supplementary Files/Supplementary Materials File Blots - (File S1).pptx]

## Slide 1
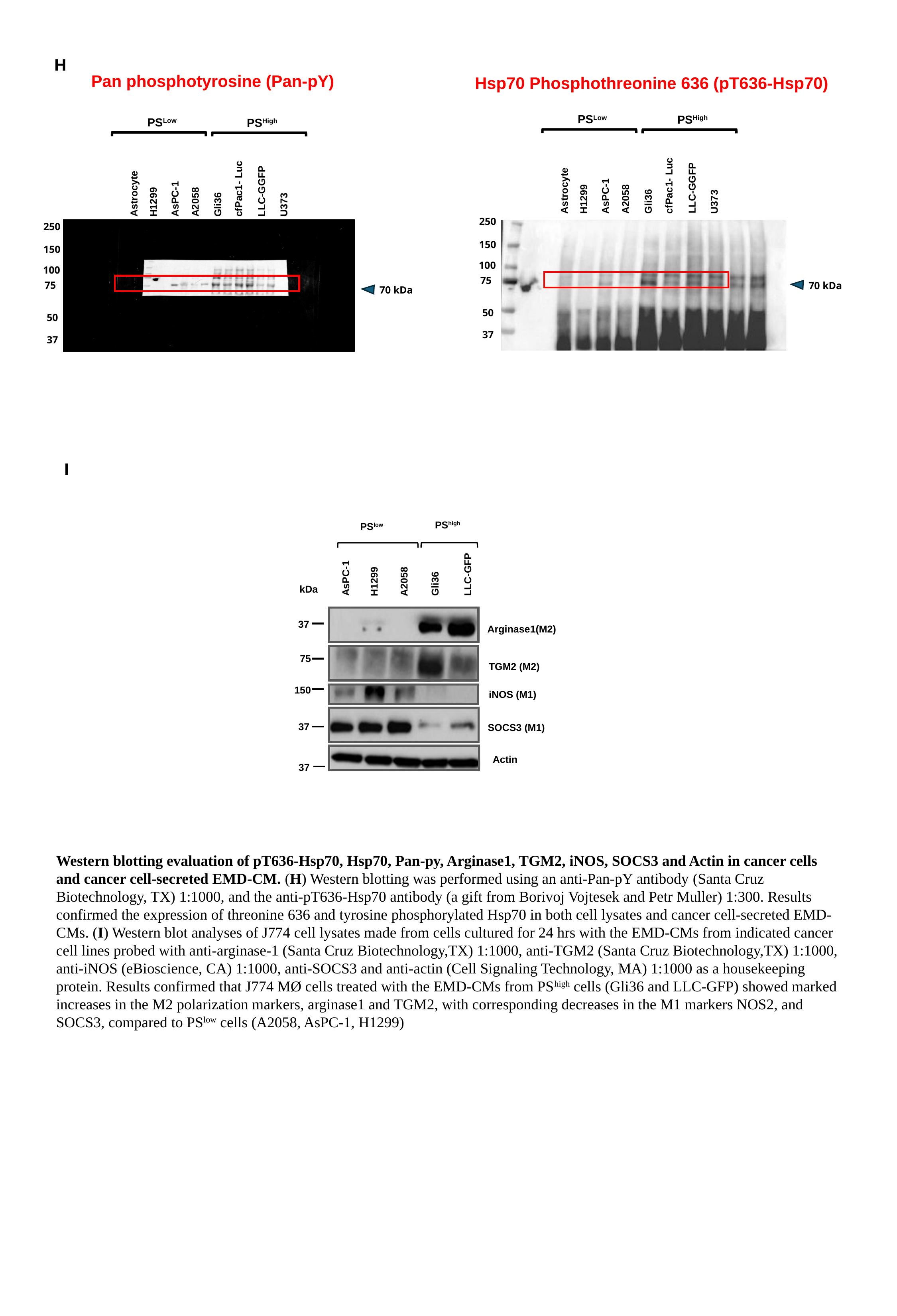

H
Pan phosphotyrosine (Pan-pY)
Hsp70 Phosphothreonine 636 (pT636-Hsp70)
PSLow
PSHigh
cfPac1- Luc
LLC-GGFP
Astrocyte
AsPC-1
H1299
A2058
Gli36
U373
PSLow
PSHigh
cfPac1- Luc
LLC-GGFP
Astrocyte
AsPC-1
H1299
A2058
Gli36
U373
250
150
100
75
50
37
250
150
100
75
50
37
70 kDa
70 kDa
I
PShigh
PSlow
LLC-GFP
AsPC-1
H1299
A2058
Gli36
kDa
37
75
150
37
Actin
37
Arginase1(M2)
TGM2 (M2)
iNOS (M1)
SOCS3 (M1)
Western blotting evaluation of pT636-Hsp70, Hsp70, Pan-py, Arginase1, TGM2, iNOS, SOCS3 and Actin in cancer cells and cancer cell-secreted EMD-CM. (H) Western blotting was performed using an anti-Pan-pY antibody (Santa Cruz Biotechnology, TX) 1:1000, and the anti-pT636-Hsp70 antibody (a gift from Borivoj Vojtesek and Petr Muller) 1:300. Results confirmed the expression of threonine 636 and tyrosine phosphorylated Hsp70 in both cell lysates and cancer cell-secreted EMD-CMs. (I) Western blot analyses of J774 cell lysates made from cells cultured for 24 hrs with the EMD-CMs from indicated cancer cell lines probed with anti-arginase-1 (Santa Cruz Biotechnology,TX) 1:1000, anti-TGM2 (Santa Cruz Biotechnology,TX) 1:1000, anti-iNOS (eBioscience, CA) 1:1000, anti-SOCS3 and anti-actin (Cell Signaling Technology, MA) 1:1000 as a housekeeping protein. Results confirmed that J774 MØ cells treated with the EMD-CMs from PShigh cells (Gli36 and LLC-GFP) showed marked increases in the M2 polarization markers, arginase1 and TGM2, with corresponding decreases in the M1 markers NOS2, and SOCS3, compared to PSlow cells (A2058, AsPC-1, H1299)
